# Posterior Reversible Encephalopathy Syndrome With Hemorrhagic Conversion in a Patient With Active Polysubstance Abuse: A Case Report and Review of Literature

**DOI:** 10.7759/cureus.30909

**Published:** 2022-10-31

**Authors:** Cassidy M Bender, Christina E Mao, Amirhossein Zangiabadi

**Affiliations:** 1 Neurology, St. George's University School of Medicine, Los Angeles, USA; 2 Stroke and Neurology, Saint Francis Medical Center, Lynwood, USA

**Keywords:** blood brain barrier, hemorrhagic conversion, methamphetamine intoxication, substance abuse, cocaine toxicity, meth, posterior reversible encephalopathy syndrome (pres)

## Abstract

Posterior reversible encephalopathy syndrome (PRES) is a neurovascular sequence noted in patients with preeclampsia/eclampsia, solid-organ/bone marrow transplantation, and malignant hypertension. The mechanism in which PRES occurs has not yet been determined. It has been hypothesized that it may be related to endothelial cell dysfunction or injury leading to the compromise of the blood-brain barrier. The clinical presentations vary but are similar to symptoms of increased intracranial pressure, such as headache, visual changes, focal neurological deficits, seizures, and altered mental status. Although the pathology suggests reversibility, that is not always the case in which severe ischemic damage has occurred. We present a patient who came to the emergency room with a history of substance abuse and tested positive on a urinary toxicology screen for methamphetamine and cocaine. In the US, polysubstance use has been more prevalent in recent years. Furthermore, literature has highlighted the additive effects on one’s blood pressure when such drugs are combined. Our patient presented with altered mental status, hypertension, and pinpoint pupils. Over the course of her stay, the patient’s mentation slowly improved and was able to follow commands intermittently. We believe that this is the first documented case of polysubstance abuse in correlation to PRES. We hypothesize that the mechanism of PRES resulted from the multiplicative effect of several illicit drugs known to cause transient hypertensive episodes and their ability to disrupt the structural proteins imperative for the blood-brain barrier.

## Introduction

Posterior reversible encephalopathy syndrome (PRES) is a clinico-radiological disorder first reported by Hinchey and colleagues in 1996. The pathophysiology is not entirely understood but has been highly correlated with hypertension and endothelial injury. This syndrome can be characterized by vasogenic edema of the subcortical white matter, particularly the parenchyma supplied by the posterior circulation [1]. Our case focuses on the clinical and neuroimaging features of PRES induced by polysubstance abuse, specifically methamphetamine and cocaine. In recent years, combining multiple recreational drugs has become more popular due to its amplified pleasurable effects. In fact, such combined drug intoxication has led to an increased number of emergency department visits in the last few years [2]. The amplified consequential product of such behavior has rarely been linked to the clinico-radiological condition of PRES.

## Case presentation

A 45-year-old African American female with a past medical history of chronic stimulant abuse and concurrent uncontrolled hypertension presented to the emergency department of an outside hospital with altered mental status, pinpoint pupils, and hypertensive urgency. She was immediately given an unknown dosage of intranasal naloxone without improvement. She was then transferred to our hospital for a higher level of care. On admission, her blood pressure was 172/119 mmHg, heart rate was 94 bpm, temperature at 97.6°F, and respiration rate at 16 breaths per minute. She appeared lethargic and withdrawn.

Upon physical examination, there were no obvious focal deficits other than altered mental status. On admission, she had a Glasgow Coma Scale (GCS) score of 11. She was able to follow commands, move all extremities, and open her eyes spontaneously; however, she could not maintain eye contact or provide a verbal response. She was not oriented to person, place, or time. It was also noted that the patient had noticeable neck stiffness upon palpation and movement. Basic laboratory results were all within normal limits except for an elevated creatine kinase above 1,300 µ/L, most likely indicating rhabdomyolysis.

**Figure 1:**
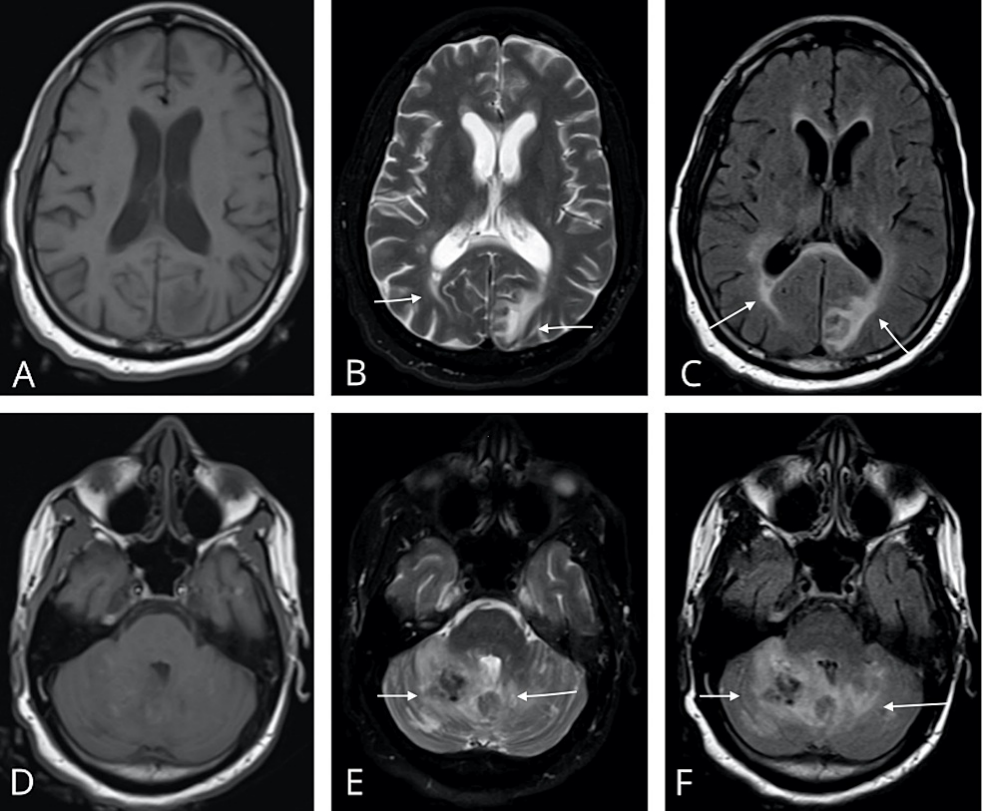
Magnetic resonance imaging of the brain in axial view demonstrating PRES in the patient. (A) T1-weighted MRI reveals no abnormalities in the cortex. (B) T2-weighted MRI reveals bilateral hyperintense regions of the periventricular and subcortical white matter of occipital lobes. (C) FLAIR sequence reveals bilateral hyperintense regions of the periventricular and subcortical white matter of the occipital lobes. (D) T1-weighted MRI reveals no abnormalities in the cerebellar region. (E) T2-weighted MRI reveals an abnormal parenchymal mass in the vermis and right cerebellar lobe. (F) FLAIR sequence reveals an abnormal parenchymal mass in the vermis with hyperintense extension into the right and left cerebellar lobe.

A computerized tomography (CT) of the head done at the outside hospital reported bilateral cerebellar encephalomalacia and left occipital lobe encephalomalacia without acute intracranial process or inflammation. These images were not obtained from the outside hospital. The patient had metal hair pins that were not easily detachable; therefore, magnetic resonance imaging (MRI) of the brain with and without contrast was delayed for three days. Once obtained, the images demonstrated multiple serpiginous enhancing lesions in the cortex and cerebellum and hemorrhagic, thickened enhancements of the cerebellum (Figures 1A-1F). It was concluded that these abnormal findings were either due to acute hypertensive encephalopathy, reversible cerebral vasoconstriction syndrome (RCVS), vascular malformation, or arteriovenous fistula. Interventional radiology conducted an intracranial angiography of bilateral vertebral and common carotid arteries, which ruled out all vascular pathologies such as arteriovenous fistula, RCVS, malformation, aneurysm, and thrombosis.

A lumbar puncture was performed to rule out meningitis/encephalitis and the results revealed a normal white blood cell count and elevated proteins. Infectious disease recommended prophylactic 1,250 mg of intravenous vancomycin, three times a day. Further cerebral spinal fluid (CSF) workup lacked evidence of infectious/inflammatory pathology. An autoimmune panel was also collected and was positive for antinuclear antibody (ANA) with a speckled pattern, which may correlate to an autoimmune disease, such as systemic lupus erythematosus or rheumatoid arthritis. Further investigation of such pathologies was out of the scope of our service’s acute neurologic care and was deferred to the outpatient clinic.

**Figure 2:**
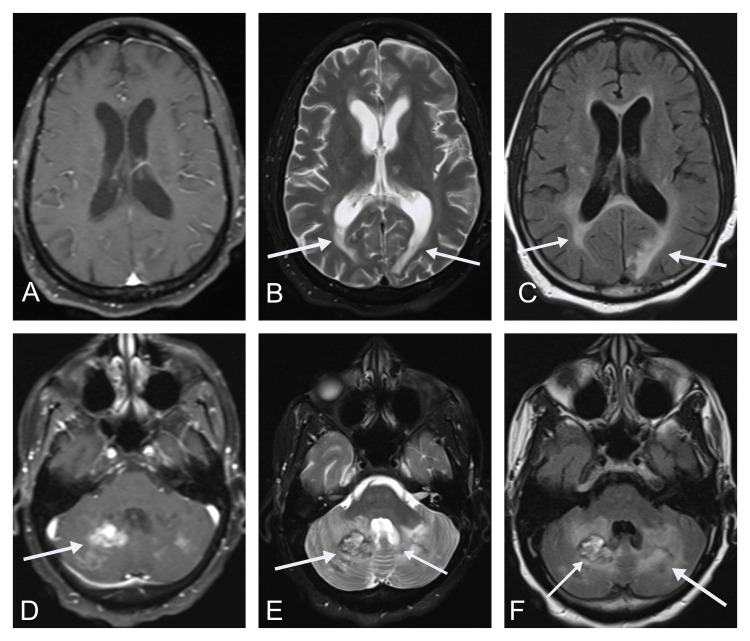
Repeat magnetic resonance imaging of the brain in axial view demonstration PRES in our patient. (A) T1-weighted MRI reveals no abnormalities in the cortex. (B) T2-weighted MRI reveals bilateral hyperintense regions of periventricular and subcortical white matter of occipital lobes. (C) FLAIR sequence reveals bilateral hyperintense regions of the periventricular and subcortical white matter of the occipital lobes. (D) T1-weighted MRI reveals hyperintense regions of the cerebellar lobes. (E) T2-weighted MRI reveals an abnormal parenchymal mass in the vermis and right cerebellar lobe. (F) FLAIR sequence reveals an abnormal parenchymal mass in the vermis with hyperintense extension into the right and left cerebellar lobe.

Blood pressure was effectively controlled with 10 mg of intravenous hydralazine as needed and daily 5 mg of oral amlodipine. A week following admission, the patient was still lethargic. She was able to open her eyes but continued to avoid eye contact. She was able to follow simple commands with redirection and nod to questions. After the MRI results supported the provisional diagnosis of PRES, 100 mg of oral modafinil was given once daily to improve mental status. Although the patient remained lethargic, her mentation had significantly improved from that of admission. It was concluded that the significant edema in the posterior thalamus, occipital, and cerebellum was the cause of the altered mental status. After the addition of modafinil, the patient was more alert and responsive but had not yet returned to her previous baseline, according to her family at the bedside. Another MRI was obtained ten days after, demonstrating hyperintensities of the cerebellar hemispheres, indicating hemorrhagic conversion. The edema appreciated on FLAIR imaging appeared to have slightly decreased, particularly at the left parasagittal occipital lobe (Figures 2A-2F). Throughout her stay, the patient was managed conservatively for hypertension, with 5 mg of oral amlodipine daily, and cerebral edema, with 4 mg of intravenous dexamethasone every six hours. The patient’s admission was prolonged for roughly one month due to placement issues. She was eventually transferred to a skilled nursing facility and referred to an outpatient clinic for follow-up.

## Discussion

Pathophysiology

The vasogenic theory is the most popular theory behind PRES. It expresses the idea that when there is a rapid elevation of blood pressure in a short period of time, the cerebral autoregulatory system fails. This autoregulatory system ensures sufficient blood flow and tissue perfusion while preventing increased intracranial pressure [3]. Specifically, vascular endothelial cells release constricting factors (e.g., endothelin-1 and thromboxane) and dilating factors (e.g., prostacyclins and nitric oxide) [4]. Chronic uncontrolled hypertension above 200 mmHg damages blood vessels irreversibly, which leads to extravasation of fluid and proteins, causing cerebral edema. Additionally, damage to arterial endothelial cells prevents the release of vasoconstriction factors, which serve as a protective mechanism against cerebral edema by restricting blood flow. This theory leaves room for further investigation as it does not fully explain why angiographic studies demonstrate hypoperfusion and vasoconstriction seen in the majority of PRES cases [5]. Furthermore, about 30% of documented cases have shown only a mildly elevated peak systolic pressure [3].

An alternative theory suggests an immunological component behind PRES. An increased systemic inflammatory response (SIRS) from pathological processes such as sepsis, autoimmune, and eclampsia can damage endothelial cells. Increased blood pressure leads to cerebral hypoperfusion due to the standard functioning autoregulation system. This episode of hypoperfusion could subsequently cause further damage to the endothelial cells, ischemic injury to the brain, and vasogenic edema [3]. Again, this theory has been refuted by several cases with no concurrent systemic inflammatory pathology. As highlighted by ongoing discussions of these two theories, it is still unclear regarding the direct cause of PRES. However, our patient presented with various pathological diseases that can contribute to both vasogenic and inflammatory theories.

Typical clinico-radiologic presentation of PRES

There has been a wide variety of clinical presentations of PRES documented. Hobson et al. examine the frequency of symptoms among 120 cases. The following presentations were identified: seizures (75%), hypertension (60%), encephalopathy (28%), visual disturbances (20%), and headache (25%) [3]. Some patients may show signs of confusion and agitation, which can last several days and are often mistaken for psychosis, drug intoxication, or psychogenic states. Many triggers can lead to PRES, such as chronic kidney disease, acute kidney injury, vascular and autoimmune diseases, immunosuppressive drugs, organ transplantation, and preeclampsia [3,6]. However, the most common predisposing factor is acute hypertension, with a peak systolic pressure usually between 170 mmHg and 190 mmHg [3].

In recent literature, there has been an increased number of reports in which there is a hemorrhagic conversion of PRES, also known as hemorrhagic transformation [7]. It is the phenomenon of blood extravasation from the intravascular space into the brain tissue. Two theories have been proposed: one, reperfusion injury following ischemia, and two, rupture of small pial vessels from elevated blood pressure and disruption of the autoregulation system [7]. In a study of 151 documented PRES patients, 15.2% of the patients had suffered from the hemorrhagic conversion on radiological imaging. Immunosuppression was one of the more prevalent comorbidities (22%) associated with hemorrhage, with eclampsia being the least associated (5%). Allogeneic stem cell bone marrow transplants showed a higher incidence (46.6%) than solid organ transplants (11.7%) [7].

As suggested in the name, PRES is commonly known to affect the posterior portion of the brain -- the parietal and occipital lobes. However, recent literature suggests the rarity of sole isolation to the posterior lobes. One study reports the radiologic presentation of 109 individuals with 115 cases diagnosed with PRES. There was a noticeable variation in the affected white matter identified: parietal/occipital lobes (94%), frontal lobe (77%), temporal lobe (64%), and cerebellum (53%) [6,8]. The same study also reported that vasogenic edema extended beyond the cerebral cortex: basal ganglia (34%) and brain stem (27%) [6,8]. MRI is the best imaging modality to identify PRES, specifically T2-weighted and T2 FLAIR (fluid-attenuated inversion recovery). However, it is essential to note differential diagnoses for PRES also have a similar hyperintense presentation. Such differentials include demyelination, infectious etiologies (meningitis, encephalitis), progressive multifocal leukoencephalopathy (PML), ischemia, and infarction. The most common presentation of PRES shows a bilaterally symmetric pattern with no restriction of diffusion. This pattern makes it easy to rule out most of the differentials previously stated since those typically present with vasogenic edema isolated to a singular vascular territory and/or an asymmetric pattern [9].

It is important to note that RCVS has similar clinical presentations that are often mistaken for PRES: headaches, seizures, stroke, and subarachnoid hemorrhage. Therefore, it is crucial to include angiographic studies to rule out multifocal areas of narrowing in cerebral arteries [10].

Illicit drugs and hypertension

Methamphetamine (Meth) is known to induce hypertension and cause vascular changes such as vasculitis through its sympathomimetic effects [11]. Its mechanism of action enhances the release of monoamines such as norepinephrine, serotonin, and dopamine. Although there is a lack of literature correlating meth use to PRES, the few cases reporting the correlation have attributed the PRES diagnosis to acute hypertension. One case report discusses a 45-year-old male who initially presented with a severe headache and systolic blood pressure of 250 mmHg after ingestion of amphetamines. The MRI showed multiple round-shaped hyperintense lesions in bilateral cerebellar hemispheres with minimal involvement of the cerebral hemisphere on FLAIR. Though an unusual presentation, it was determined that the most likely diagnosis was PRES in relation to the patient's amphetamine abuse [11].

It has also been proposed that meth disrupts the blood-brain barrier (BBB) through two mechanisms. Firstly, meth plays a role in downregulating subunits of critical structural proteins of the BBB, such as occludin, claudin-5, and zona occludens. Secondly, meth-induced peripheral organ damage can result in the disruption of the BBB. For example, damage to the liver can lead to elevated circulating ammonia, which has known oxidative, excitotoxic, and inflammatory consequences leading to the compromise of the BBB [12].

Cocaine is a commonly abused drug with sympathomimetic effects. Its known mechanism of action is to block the reuptake of monoamines such as serotonin, dopamine, epinephrine, and norepinephrine. Cocaine-induced PRES is also rarely documented, as with meth. However, it has been repeatedly shown to cause an acute elevation in blood pressure due to excess monoamines. One case report discusses a 40-year-old female who had relapsed from cocaine after three months of sobriety. She also had comorbid chronic kidney disease and uncontrolled hypertension. Her documented blood pressure on admission was 189/140 mmHg, with several subsequent systolic blood pressure readings above 200 mmHg. She complained of a frontal headache, photophobia, nausea, and vomiting lasting several days. She fell asleep several times during the examination but was easily aroused when touched. She denied any neurological deficits, fever, nuchal rigidity, seizures, or visual disturbances. CT on admission showed hypodense abnormalities in the left occipital, left parietal, and right thalamus. FLAIR and T2 weighted MRI showed increased signals in the brainstem, thalamus, and cerebellum. She was diagnosed with PRES and promptly treated with IV antihypertensives, which relieved the admitting symptoms within two days of treatment [6].

Cocaine and methamphetamines have different mechanisms of action, but both result in stimulating the sympathetic nervous system. Cocaine has a much shorter half-life than methamphetamines, often leading to the combination of the two in search of a more prolonged and intense “high” [13]. The consequential additive effects are not taken into consideration during a euphoric state. Tachycardia, arrhythmia, seizures, hypertension, and overdose are a few of the more life-threatening effects. It is proposed that stimulant-induced PRES may have a higher incidence rate than reported due to CT being the more common radiological modality used in the emergency department [6].

## Conclusions

The cause of PRES is multifactorial, as presented in our patient. She has a history of chronic uncontrolled hypertension, chronic stimulant abuse, and a probable autoimmune disease, all possible contributing factors to PRES. Although we cannot document direct causation, several cases have reported the correlation between acute hypertensive episodes from stimulant substance abuse and PRES and, subsequently, hemorrhagic conversion. More research must be conducted to understand this rare clinico-radiological disease. Discovering the exact pathophysiology may help develop therapeutic treatment options to prevent irreversible damage in the future.
